# Physiological synchrony and shared flow state in Javanese gamelan: positively associated while improvising, but not for traditional performance

**DOI:** 10.3389/fpsyg.2023.1214505

**Published:** 2023-08-17

**Authors:** Hannah Jennet Gibbs, Anna Czepiel, Hauke Egermann

**Affiliations:** ^1^York Music Psychology Group, Music, Science and Technology Research Cluster, School of Arts and Creative Technologies, University of York, York, United Kingdom; ^2^Department of Music, Max Planck Institute for Empirical Aesthetics, Frankfurt, Germany; ^3^Department of Neuropsychology and Psychopharmacology, Faculty of Psychology and Neuroscience, Maastricht University, Maastricht, Netherlands; ^4^Institute for Music and Musicology, TU Dortmund University, Dortmund, Germany

**Keywords:** flow state, gamelan, synchrony, coupling, skin conductance, heart rate

## Abstract

The experience of shared flow refers to the optimal balance between challenge and ability for a given task, resulting from interpersonal action in a group situation. The performance of Javanese gamelan is an ideal setting to investigate shared flow, due to the requirement that all performers on varying instrumental parts work harmoniously, allowing for shared flow and its native equivalent, *ngeli*. To minimise the disruption of flow, while still measuring it continuously, one way to assess a person’s state is by measuring physiological responses of the sympathetic (i.e., fight-or-flight) system, namely heart rate and skin conductance. Flow has been related to physiological signatures, and shared actions in music-making have been related to synchronised physiology. However, to our knowledge, no study yet has directly investigated the links between shared physiology and shared flow. Therefore, this study aimed to assess the associations between flow states, physiological synchrony, and Javanese gamelan playing. Subsequently, we tested for differences between advanced and beginner groups playing traditional gamelan pieces and improvising. Firstly, a factor analysis revealed a two-factor solution of Awareness and Absorption for self-reported shared flow. Next, using inter-subject correlation to assess synchrony and circular shuffling to infer significance, we found a greater proportion of significance in traditional playing compared to improvised playing for the experienced group, and the opposite for the beginner group. Lastly, linear mixed models revealed largely positive associations between synchronised physiology and shared flow during improvised playing, and negative associations during traditional playing, regardless of experience levels. This study demonstrates methodological possibilities for the quantitative study of shared flow in music-making contexts, and potential differences in shared flow experience in improvised and traditional, or prescribed, playing.

## 1. Introduction

The underlying mechanics of musicians performing together are somewhat mysterious. In gaining insights into this phenomenon, a growing body of empirical research has shed light on the behaviours, interactions, and coordination strategies of ensemble musicians (Gugnowska et al., 2022; Khalil et al., 2022; Kohler et al., 2022; Delius and Müller, 2023). One mechanism which could aid collaborative musical contexts is the experience of shared flow, though this has been seldom explored (Cochrane, 2017; Harmat et al., 2021; Tay et al., 2021). Shared flow refers to flow as a result of interpersonal action in a group situation (Pels et al., 2018).

### 1.1. Flow and shared flow

To date, flow states have most frequently been investigated in individuals and are defined primarily as a balance between challenge and skills for a given task and moderate mental effort among other facets (Nakamura and Csikszentmihalyi, 2014).

Initial conceptions of shared flow stemmed from Csikszentmihalyi’s conception of individual flow (Csikszentmihalyi and Larson, 1987; Csikszentmihalyi and Csikszentmihalyi, 1988). This later inspired Sawyer’s work on group flow and group creativity (Sawyer, 2006), who argued that group flow goes beyond the sum of independent flow experiences. For some researchers, shared flow or group flow is often seen as an extension of individual flow state, where it typically arises through and contributes to synchronous or coordinated action (Gloor et al., 2013; Walker, 2021), or challenging cooperative activity (Magyaródi et al., 2022). However, recently it has been argued that different forms of shared flow exist and vary according to the level of self-other overlap, or the level of interconnectedness and closeness between one individual and another. Hackert et al. (2022) describe fundamental differences between what they call ‘shared interactive flow’ and ‘group flow’, whereby the degree of self-other overlap can be thought of as a spectrum that may vary between these two flow states; higher overlap is the latter, while less overlap is the former. Both shared interactive flow and group flow may occur in alternation, due to the dynamic and changeable nature of group flow (Pels and Kleinert, 2022). Shared interactive flow is focused on the shared task, although is experienced on an individual level within an interactive social context of moderate self-other overlap. In group flow, however, the focus is both on the task and the group due to the continuous and synchronous social interaction being a requirement, and self-other overlap is at its fullest. Just as group flow involves a greater merging of self and other, so too does moving in synchrony (Hu et al., 2022), or coordinating with one another in a musical group (Liebermann-Jordanidis et al., 2021). Self-other overlap is typically measured *via* the visual scale of Inclusion of Self in Other scale (Aron et al., 1992), however, we simply interpret this here as the hypothetical level of connectedness present among a group.

Musical situations are one paradigm where the dynamism of flow states can be experienced and studied in a multitude of ways, as music is often played in an ensemble where there is a coordinated group goal to perform music (see reviews, Tan and Sin, 2021; Tay et al., 2021). Situations in which the actions of one influence another as a series of causes and effects, for example, improvisation, are seen as most favourable for group flow to arise. Additionally, an individual’s performance may change over time. The musical material may vary in the level of challenge it presents, and so too might the individual’s capacity to meet such challenge. It is often exemplified through performance in improvising jazz groups, whereby actions of individuals are highly interrelated across the group, and the resulting flow is therefore above any one player due to the social, dynamic and collaborative context in which it arises (Hackert et al., 2022). In such contexts, individual flow experiences are difficult to disentangle from group flow experiences, due to the social context being the catalyst for flow. This article adopts the understanding that there may be distinctions between shared interactive flow and group flow (Hackert et al., 2022) between playing contexts, for instance between improvised and prescribed playing, which may also depend on the level of musical experience. Consequently, these terms are used in relation to their relevant contexts. Shared flow is used as a more general, overarching term.

### 1.2. Relevance of flow state to gamelan

Shared interactive flow and group flow are likely mechanisms to aid music ensemble performances. While there are plenty of different kinds of ensembles that could be examined, the current study focused on an ensemble type that affords greater opportunities for flow states, namely Javanese gamelan. Javanese gamelan requires that all individual instruments work harmoniously, allowing for the potential occurrences of shared interactive flow or group flow, and the native equivalent, *ngeli*, meaning to float together (Tan et al., 2020). Underpinning the egalitarian ethos of gamelan is interlocking synchrony of structural instruments and repetitive cyclical patterns, whereby governance is distributed throughout the ensemble in an algorithmic sense, and individual and combined outcomes are reciprocally predicted and monitored (Matthews, 2018). As opposed to the case of a Western orchestra, where the first violin or conductor may act as lead roles, gamelan is non-hierarchical in that all instruments are of equal significance (Sorrell, 1990).

Most types of gamelan cross-regionally share the same fundamental basis: a core melody, punctuation of the melody, and drumming patterns (Loth, 2016). Although there are some differences between gamelan practises specific to different regions, its conceptual basis of meaning and structure varies minimally (Loth, 2014). In traditional pieces or *gendhing*, the core melody, or *balungan* line, consists of groups of 4 beats, known as *gatra*, which is typically taught aurally or through prescribed written notation (Sorrell, 1990). A *gendhing* incorporates cycles of series of *gatra* in which melodic parts play the continuous *balungan* line, and other structural instruments play interlocking beats punctuating the structure (Pickvance, 2005). Generally, a drummer typically leads the group from the start to the end of pieces, signalling changes in tempo and section (Macdonald et al., 1999). The structural instruments are not as technically demanding as the drums, melodic or solo instruments, yet these parts are equally important in their role of signalling subdivisions of the structure for the rest of the players. The end of each cycle is marked by a gong, which also acts as a signifier to return to the start. This cyclical, and inevitably, repetitive nature of gamelan allows for effortless memorisation and full absorption for individuals in the activity (Diamond, 1979), and in turn, shared flow state.

Although gamelan has these general structures as described above, we look at two overall styles of gamelan: playing from a traditional piece and improvisation. Within traditional pieces, structural and temporal changes require individuals to think beyond their individual roles. For instance, how a change in drumming pattern might trigger rhythmic subdivisions for certain instruments, and how a change in register for those same instruments might trigger a change of section and melodic material for the entire ensemble. Such structures and ensemble dynamics may allow for the potential rise and fall of flow through the fluctuations in shared attention. Therefore, in the context of traditional gamelan, the actions of one influence another, spreading throughout the ensemble. Where traditional pieces are often written down, players may focus primarily on their individual roles, and intermittently be forced into an awareness of the changeable interactive setting. This may subsequently facilitate shared interactive flow, as this is related to individual flow experience of flow in an interactive setting, whereby individuals are focused on their tasks, or individual parts and their interaction with others (Hackert et al., 2022). Improvised playing, on the other hand, involves a great degree of self-other merging and shared awareness, and therefore lends itself more to group flow due to the entire focus being on the shared task as a result of the social interaction. Findings from Liebermann-Jordanidis et al. (2021) may support this, as they found interpersonal coordination to be facilitated by simultaneous self-other segregation and integration, allowing for performers to adapt to and anticipate the actions and timings of others. Improvisations are not so typical in traditional gamelan ensembles. However, there are exceptions for instruments requiring a greater level of skill, where elaborations around the melody tend to have a degree of improvisatory character (Perlman, 2004). Furthermore, gamelan lends itself well to group improvisation. Indeed, improvisationis a prevalent technique in both gamelan-based music therapy and community music groups in the United Kingdom, as the instruments are tuned to scales that allow for harmonies which form a cohesive sound with ease (Loth, 2014, pp. 113–114).

Depending on individual ability and level of group training, which is seen to be paramount to fostering group flow experiences (Salanova et al., 2014; Pels et al., 2018), a highly skilled gamelan group may reach group flow in playing a traditional piece, so long as all individuals were extremely familiar with their individual parts, which may not be the case with beginner groups. This is also supported by an assertion by (Sawyer, 2015) that group flow is more likely to occur when all agents are equally involved in the creative process, and consequently, group flow may be unachievable if players do not have comparable skills.

### 1.3. Measurement of flow

#### 1.3.1. Self-reports

Many studies on shared flow centre on qualitative investigation through means of phenomenology or grounded theory (Hart and Di Blasi, 2015; Hill et al., 2018) or retrospective pre-validated scales. In measuring flow quantitatively, the Flow State Scale or Flow State Scale-2 (Jackson and Marsh, 1996; Jackson and Eklund, 2002), for instance, have been used to assess the presumed level of shared flow by equivocating the individual’s experience to that of the group (Keeler et al., 2015; Gaggioli et al., 2017). Alternatively, scales designed to measure the shared flow experience rather than the individual could be adopted and validated in musical contexts, according to Tay et al. (2021). One suggestion is the group task absorption scale by Salanova et al. (2014). However, the Shared Flow Scale by Zumeta et al. (2016) might be a better tool. Although this tool does not align with the theory of Hackert et al. (2022) nor does it differentiate between the potential experiences of shared interactive flow and group flow, it yields greater promise. First, it is based on the flow items posited by Jackson and Marsh (1996) and replaces individual pronouns with plural. Second, it was validated in the context of a drumming march.

A self-report measure may not be enough to disentangle shared interactive flow from group flow. However, one potential way of viewing the differentiation is by considering Hackert et al. (2022) assertion that group flow may be measurable from a third-party observation or objective measure, even when not evident from an individual perspective. A continuous and undisruptive measure may therefore be employed alongside the Shared Flow Scale, such as physiological measures.

#### 1.3.2. Physiological indicators of flow state

Flow states have been said to co-activate both branches of the autonomic nervous system; the sympathetic branch, linked to the fight-or-flight response, and the parasympathetic branch, linked to rest and digestion (Tozman et al., 2015).

Much of the work on physiological indicators of flow state has primarily assessed Heart Rate Variability (HRV) which has been associated with domains of flow linked to concentration or balanced challenge and skill. This has been observed through a linear relationship with parasympathetic activation (Peifer et al., 2014; Tozman et al., 2015; Bian et al., 2016; Thissen et al., 2021), and moderate sympathetic activation (de Manzano et al., 2010; Peifer et al., 2014). Taken together, these findings form a varied understanding of how cardiovascular activity might be related to flow, but generally, it is agreed that it is indicative of co-regulation of sympathetic and parasympathetic activity (Ullén et al., 2010; Tian et al., 2017; Thissen et al., 2021), potentially in the form of an inverted-u-shape (Peifer et al., 2014; Bian et al., 2016). Such studies indicate the substantial potential of HRV to measure potential flow experience. However, the necessity to calculate such variance over at least 5 minutes (Task Force of the European Society of Cardiology the North American Society of Pacing Electrophysiology, 1996) does not allow for the ability to observe momentary changes in parasympathetic and sympathetic activation. While HR changes as indicators of flow activity may be confounded by physical exertion (Jaque et al., 2020), there still seems to be potential to relate heart rate (HR) changes to changes in flow experience in the context of music performance (Horwitz et al., 2021; Jha et al., 2022). Ultimately this is due to the potential for sympathetic and parasympathetic activity to increase or decrease HR, respectively (Pérez et al., 2021).

In addition to cardiovascular activity, skin conductance (SC), is also prevalent in studies on physiological responses and flow state due to it being indicative of arousal in the sympathetic nervous system (Bian et al., 2016), and flow requiring a moderate level of arousal between boredom and stress (Csikszentmihalyi and LeFevre, 1989; Peifer et al., 2014). Supporting this notion, Tian et al. (2017) found moderate levels of SC as being related to flow experience compared to that of stressful states. SC changes have also been linked to mental effort associated with planning and execution in piano improvisation (Dean and Bailes, 2015), which could, in turn, suggest an important role for SC in the study of flow in dynamic music performance.

While the outlined studies show relationships between individual flow states, we move to consider how shared flow experiences may be reflected by shared physiological patterns. One study that attempted to assess the relationship between these variables in chamber musicians was conducted by Horwitz et al. (2021), however, no significant relationship between synchronised flow experiences and overall HR means was observed. As shared flow experiences often encompass synchronised movements (Gloor et al., 2013), and self-other overlap are related more specifically to group flow (Hackert et al., 2022), it is relevant to also consider physiological parameters related to these facets specifically.

Self-other overlap, crucial to the presence of shared interactive flow and group flow, is related to social bonding, perspective-taking, cooperation and coordination (Galinsky et al., 2005), all of which have been related to coupling of physiological measures (Vanutelli et al., 2017; Gordon et al., 2020; Tomashin et al., 2022). Physiological coupling of both SC and cardiac activity in dyadic interactions or groups has been noted in numerous contexts, typically *via* methods of inter-subject correlation (ISC) of time-series data. HR has been found to synchronise in response to shared cognitive processing of video stimulus (Madsen and Parra, 2022), and audio narratives (Pérez et al., 2021), but also in response to co-ordinated action in group drumming (Gordon et al., 2020) and togetherness in improvised movement (Noy et al., 2015). Tomashin et al. (2022) found that physiological synchrony could predict group cohesion, resulting from both levels of coordination in drumming and a group decision-making task. Coupling of both SC and RR has been found to occur across audiences of live concerts (Czepiel et al., 2021), and in group decision-making tasks (Gordon et al., 2021). Furthermore, HRV has been found to synchronise across individuals (Ruiz-Blais et al., 2020; Lange et al., 2022). Nonetheless, to our knowledge, there has yet to be any study of ISC of physiological continuous signals in music performance contexts, nor has there been any insight into how shared flow might be reflected in shared physiology.

### 1.4. Aims

The synchronised, interlocking, repetitive, and egalitarian aspects of gamelan described yield an optimal opportunity to disentangle shared interactive flow and group flow. Self-reported measures in flow research are often used to assess an individual’s perceived shared flow experience, but in group flow, this is more likely to be assessed from an objective measure (Hackert et al., 2022). We assess this through a combination of self-reports on flow and physiological measures. Here, the assumption is that a greater sense of collectivism or cohesion would lend itself to greater physiological togetherness (Gordon et al., 2020; Tomashin et al., 2022) and in turn potential group flow. Given the outlined research gap in clarifying the distinctions between shared interactive flow and group flow, and measuring physiology in group performance contexts, we predict potential associations between group flow (rather than shared interactive flow) and physiological coupling. Ultimately, this is due to an optimum paradigm for group flow being when individuals are working together on a task involving great interactivity and interdependence, resulting in the highest level of interpersonal connectedness.

Since coordinated action, such as in musical ensembles, is associated with synchronised physiological responses (Ruiz-Blais et al., 2020; Hoehl et al., 2021), we were interested in whether such an effect emerges more in a setting facilitating group flow (improvised playing) than in shared interactive flow (traditional playing). There is a need to extend our understanding of physiological synchrony through quantitative naturalistic experimental designs (Chabin et al., 2020; Tervaniemi, 2023), and the outlined literature indicates links between physiological mechanisms and flow states. While the one hand, individual flow has been related to physiological signatures (de Manzano et al., 2010; Thissen et al., 2021), and on the other, shared actions are related to synchronised physiology, to our knowledge, no study yet has directly investigated these links between shared physiology with shared interactive flow and group flow. As Pels et al. (2018) recommended, studies should investigate conditions under which group flow may occur. Consequently, this study aimed to test whether there are associations between synchrony of physiological measures, individual and shared flow states; in addition, whether traditional or improvised playing as well as the level of experience with playing gamelan have any influence on these potential associations.

The following research questions will therefore be considered:

*RQ 1* Is the Shared Flow Scale in gamelan playing valid and reliable in its proposed factor structure?

*RQ 2* Is there a difference in significantly correlated windows of physiological synchrony between participants when playing traditional gamelan music compared to improvising as a group, and does this differ between levels of expertise?

*RQ 3* How does self-reported shared flow relate to average physiological synchrony in traditional and improvised playing?

## 2. Materials and methods

### 2.1. Participants

Participants with varying experience of playing gamelan were invited for one of three recording sessions. The first recording session consisted of participants with prior experience of gamelan playing recruited from the current members and tutors of Gamelan Sekar Petak, a gamelan group based at the University of York, United Kingdom (*N* = 13, age M = 29.6, SD = 10.2, 61.5% female). The second and third recording sessions consisted of participants with no, or very minimal, prior experience of playing gamelan (*N* = 16, age M = 24.75, SD = 5.58, 62.5% female) and were students recruited from the University of York (mostly from the music department). This latter group was divided into two equal-sized groups for two recording sessions. For both groups, players’ instrumental parts encompassed different instrumental groups: structural instruments of *gongs* and *kempuls* (shared by one player in the beginner group and played by two players in the advanced group); *kethuk* and *kenongs* (similarly shared by one player in the beginner group, and played by two players in the advanced group); *bonang barung* and *bonang panerus* parts; and metallophone *balungan* instruments of *sarons, slenthem and demung.* The advanced group included: *kempyang,* played alongside the *kethuk* by the same player; an added *peking*, a slightly more elaborating *balungan* instrument; *kendhang* and *ciblon,* a set of drums playing intricate patterns that use a variety of techniques; and *gender*, a complex metallophone playing elaborate patterns weaving around the *balungan* line with both hands.

### 2.2. Procedure

Both experience groups played two pieces: one traditional gamelan piece from central Java that was selected to suit their collective ability and were asked to improvise a piece as a group spontaneously. The advanced group played *Ladrang Pangkur Pelog Pathet Barung* (12 min and 56 s), which had been rehearsed for several months in preparation for a concert and a dance performance. The beginner groups were taught the principles of gamelan playing and learnt their traditional piece the same morning under the guidance of a tutor, which was *Lancaran Baita Kandas Pelog Pathet Nem* (5 min and 5 s for the first beginner session, and 5 min and 51 s in the second beginner session due to an additional repetition of the opening cycle). *Baita Kandas* was largely learnt aurally due to the unfamiliarity of gamelan notation for beginners, while many players of *Pangkur* relied on the notation to varying degrees. Little instruction was given on how to navigate improvisations; players were simply asked to leave space for others, not feel pressured to play constantly and be mutually responsive to one another. Before the session, and after playing both the traditional and improvised pieces, participants completed questionnaires (detailed below), while physiological measures were taken throughout the performances. The entire sessions were video recorded. The recording sessions, including the set-up of devices, lasted around 90 min each. Due to measurement error, physiological data from two participants for each group was removed completely (total *N* = 25, experienced *N* = 11, beginners *N* = 14).

### 2.3. Measures

#### 2.3.1. Self-report measures of flow state

As the study was exploratory, and to our knowledge the first of its kind, a wide variety of pre- and post-experiment measures were taken. Several of these measures encompassed items relating to the nine dimensions of flow state theorised by Csikszentmihalyi (2000):

1. Balance between the challenges of and the related individual skills for a given task.
2. Clear goals for the task.
3. Unambiguous, ongoing feedback on the progress of task accomplishment.
4. Concentration on the task at hand.
5. Merging of action and awareness.
6. Loss of self-consciousness.
7. A sense of control.
8. Transformation of time.
9. An autotelic experience.

Although we used the Dispositional Flow Scale (DFS-2), and the CORE Flow State Scale (Martin and Jackson, 2008), this paper just focuses on the experience of shared flow, using the Shared Flow Scale (SFS), a 27-item 5-point likert scale (Zumeta et al., 2016). This scale is based on a Spanish version of the original DFS (Jackson and Marsh, 1996) and an adaptation (Calvo et al., 2008), in line with the nine dimensions of flow outlined above. It assesses the experience of flow after participation in an activity, and rather than assessing the individual experience as the DFS-2 does, it instead assessed shared flow by replacing singular personal pronouns of ‘I’ with ‘we’. Due to the exploratory and multi-faceted interests of our study, several other measures were also attained, including the Goldsmiths-Musical Sophistication Index (GOLD-MSI Müllensiefen et al., 2014), the Positive and Negative Affective Schedule (PANAS) (Watson et al., 1988), the Perceived Emotional Synchrony Scale (PESS) (Wlodarczyk et al., 2020), and the Warwick-Edinburgh Mental Wellbeing Scale (WEMWBS) (Tennant et al., 2007). However, as this paper and associated research questions are focused solely on the intersections of shared flow and physiological synchrony we do not include these in our analyses here.

#### 2.3.2. Physiological measures

Skin conductance (SC) and electrocardiogram (ECG) measures were recorded using Shimmer Sensors, where measurement areas for sensor electrodes were prepared using 70% alcohol swabs. ECG was measured *via* a four-lead configuration from the chest. From these measures, heart rate (HR) and skin conductance (SC) were extracted to reflect an individual’s physiological arousal. SC was measured *via* the inside of the right foot, which is a comparable anatomical recording site to that of the typical hand configuration (Sanchez-Comas et al., 2021; Hossain et al., 2022). This method was also selected due to having greater ecological validity, as it allows for the participant to play gamelan freely with both hands and because players traditionally remove outer footwear regardless as a way of showing respect to the instruments.

### 2.4. Data analysis

#### 2.4.1. Physiological pre-processing

Physiological data, recorded at 256 Hz, were pre-processed using custom-made scripts for MATLAB. ECG data were pre-processed using a Butterworth bandpass filter with 0.2 and 12 Hz cutoff points, and 4th order zero-phase filtering. We extracted the ECG from the standard lead II configuration (Golland et al., 2015; Gordon et al., 2021). Following this, inter-beat intervals (IBIs) were calculated from the peaks of the ECG. Continuous HR was then calculated from the IBIs using the ‘interp1’ MATLAB (with the ‘nearest’ method specified) and lowpass filtered at 0.05 Hz. Only HR values between 40 and 140 beats per minute, typically associated with healthy resting HR for adults, were used in subsequent analyses. This step was taken to ensure the exclusion of any inaccurate data resulting from measurement error (*N* = 3). SC data were pre-processed using Butterworth lowpass filtering with a 0.5 Hz cutoff point and any further signals observed to be inaccurate were discarded (*N* = 7) The whole SC signal was used in all analyses. This resulted in *N* = 9 SC and *N* = 10 HR remaining signals for the advanced group, and *N* = 9 SC and *N* = 12 HR remaining signals for both beginner groups combined.

#### 2.4.2. ISC of physiological signals and statistical significance

Both HR and SC signals were segmented into musically meaningful bins per bar, or *gatra* in Javanese terminology. A *gatra* typically encompasses a set of four notes for the *balungan* or melody line, which forms a part of a whole cycle, often in multiples of four or eight punctuated by structural instruments, and repeated.^1^ Gamelan music is usually analysed in terms of these four-note *gatra* (Pickvance, 2005), and in this context, a *gatra* ranged from three to a maximum of 7 seconds in length depending on the tempo of the section. For the improvisation, the signals were segmented into bins of equivalent duration to that of the traditional pieces, of around 3 seconds. Physiological synchrony was obtained from instantaneous SC and HR signals with inter-subject correlation (ISC) analyses. We calculated Pearson’s r values between all possible dyads within groups, within windows of 8 *gatra* overlapping by 4, which was decided on due to the gamelan cycles in the pieces selected working largely in structures of 8 *gatra* which are repeated. The ISC procedure follows that of Pérez et al. (2021) and was calculated using their provided MATLAB scripts. Fisher’s z transformation was applied to each dyadic ISC for each segment, before calculating the mean for each set of dyadic combinations per subject. Following this, the inverse Fisher’s z transformation was applied.

Statistical significance of ISC was assessed by comparing ISC calculated with original, time-locked data with control data that was computed using circularly shifted segment shuffling. This yielded a control ISC for each dyad, repeated for each group for both HR and SC. The filtered continuous signal for each subject and section was circularly shifted by a random amount 10,000 times, producing control signals for each subject and section. The same ISC procedure was followed as above. Significance was determined by calculating the proportion of control ISCs above the threshold of the actual ISC for each subject, in both traditional and improvisational pieces.

For RQ1, we conducted a confirmatory factor analysis of the original Shared Flow Scale (Zumeta et al., 2016), followed by an exploratory factor analysis and confirmatory factor analysis to assess whether there was a more appropriate solution for the observed data. For RQ2, we observed points of significance across all pieces visually, before conducting Fisher’s exact tests to determine whether the proportion of significant ISCs differed between traditional and improvised playing, and between experience groups. Lastly, for RQ3, overall ISC-SC and ISC-HR variables were calculated by averaging all ISC across sections, to produce a global ISC average across the piece per participant, ISC-SC_mean_ and ISC-HR_mean_. This was calculated for traditional and improvised pieces separately. Linear mixed models these for each participant to analyse potential relationships between physiological synchrony and self-report shared flow factors.

## 3. Results

### 3.1. Is the shared flow scale in gamelan playing valid and reliable in its proposed factor structure?

Confirmatory factor analysis was conducted for all 27 items of the Shared Flow Scale (Zumeta et al., 2016) across the 29 participants, to test whether the original model of nine dimensions was a reasonable fit for the observed data. This original model was not admissible, as the covariance matrix of latent variables was not positive definite.

**Figure 1:**
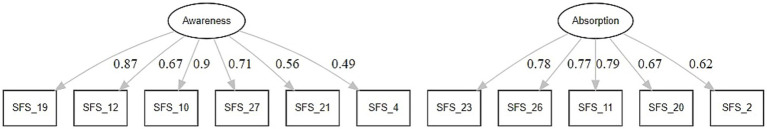
Factor loading plot showing standardised estimates of the two factors labelled *Awareness* and *Absorption*.

Therefore, we wanted to see whether there was another more appropriate solution for the data. Exploratory Factor Analysis (EFA) was then conducted using parallel analysis based on factor analysis, a maximum likelihood estimation method, and oblimin rotation. This resulted in a two-factor solution. Confirmatory factor analysis (CFA) was repeated on the model provided by the EFA, using the highest loading items and a maximum likelihood restricted estimation. This confirmed a two-factor solution of 11 items with a reasonable fit for the observed data, given the small sample size, X^2^ (43, N = 29) = 58.790, CFI = 0.906, TLI = 0.880, robust RMSEA = 0.102, SRMR = 0.095. Cronbach’s α for this solution was given at 0.854 and 0.835, demonstrating very good internal consistency for each factor. This model is outlined in Figure 1, and parameter estimates are given in Table 1. Labels of *Awareness* and *Absorption* were given to each of the factors, to reflect items that encompassed elements of flow holding a great deal of awareness, in comparison to those that were absorbing and automatic. Average variance extracted (AVE) = 0.581 for *Awareness*, and AVE = 0.536 for *Absorption*, while the covariance between the factors was 0.612. For further analysis, composite scores for each of these factors were taken.

**Table 1:** Confirmatory factor loadings for SFS factors.

Factor		Item	Estimate	Std. Err	z-value	*p*
Awareness	SFS_19	“We felt we were competent enough to meet the high demands of the situation.”	0.867	0.209	3.504	<0.001
	SFS_12	“We knew clearly what we wanted to do.”	0.668	0.080	5.240	<0.001
	SFS_10	“Our abilities matched the high challenge of the situation.”	0.904	0.205	3.544	<0.001
	SFS_27	“The group experience left us with a good impression, a good taste.”	0.708	0.211	2.421	0.015
	SFS_21	“We knew what we wanted to achieve.”	0.559	0.086	3.602	<0.001
	SFS_4	“It was really clear to us that we were doing well.”	0.494	0.082	3.175	0.001
Absorption	SFS_23	“We felt totally absorbed by what we were doing.”	0.776	0.195	2.934	0.003
	SFS_26	“We felt like time stopped while we were performing.”	0.766	0.158	5.155	<0.001
	SFS_11	“We felt that things were happening automatically.”	0.785	0.126	4.226	<0.001
	SFS_20	“We performed automatically.”	0.669	0.244	2.326	0.020
	SFS_2	“We were doing things spontaneously and automatically.”	0.616	0.127	3.216	0.001

*N* = 29.

Data from two participants were removed for all subsequent analyses due to anomalous extreme values that could not be accommodated in further model specifications.

### 3.2. Is there a difference in significantly correlated windows of physiological synchrony between participants when playing traditional gamelan music compared to improvising as a group, and does this differ between levels of expertise?

**Figure 2:**
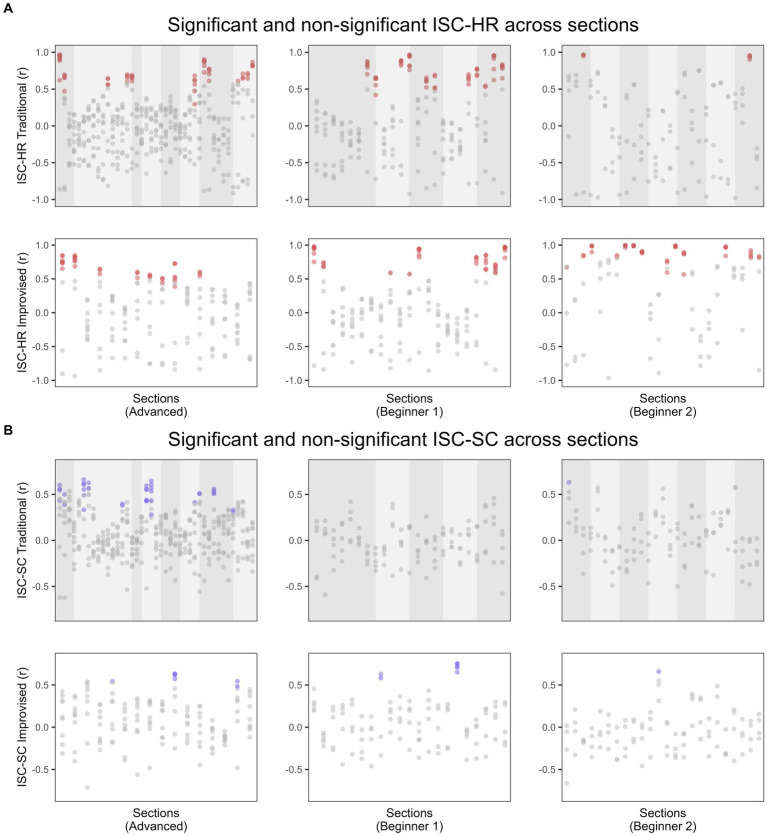
**(A)** ISC-HR and **(B)** ISC-SC computed for each participant and each section across traditional and improvised gamelan playing. Significance is denoted via coloured dots, using FDR of 0.05. Shaded regions indicate changes in musical material (i.e., where the section changes or more elaborating material is added) or tempo changes.

To test for significant ISC of instantaneous SC and HR signals, pairwise ISCs for each participant were computed across 8 *gatra* sections for traditional pieces and an equivalent section in length of 24 s for improvised pieces, before averaging across pairs to produce one ISC-SC and one ISC-HR value per participant and section. These values were then compared to values resulting from randomly shuffled signals. Figure 2 displays these ISC values across each of the playing sessions, whereby statistically significant values are denoted *via* coloured dots. This shows that sections yielding significant ISCs are observed to some degree across both playing conditions and for both SC and HR, albeit more so for ISC-HR than for ISC-SC.

Fisher’s exact tests were used to assess differences in the proportion of significant to non-significant values between the two levels of experience, with both beginner recording sessions combined, and between playing conditions. Significance here was determined by measuring the proportion of synchrony values calculated from the original data compared to control data attained through circular shuffling, using FDR of 0.05.

**Figure 3:**
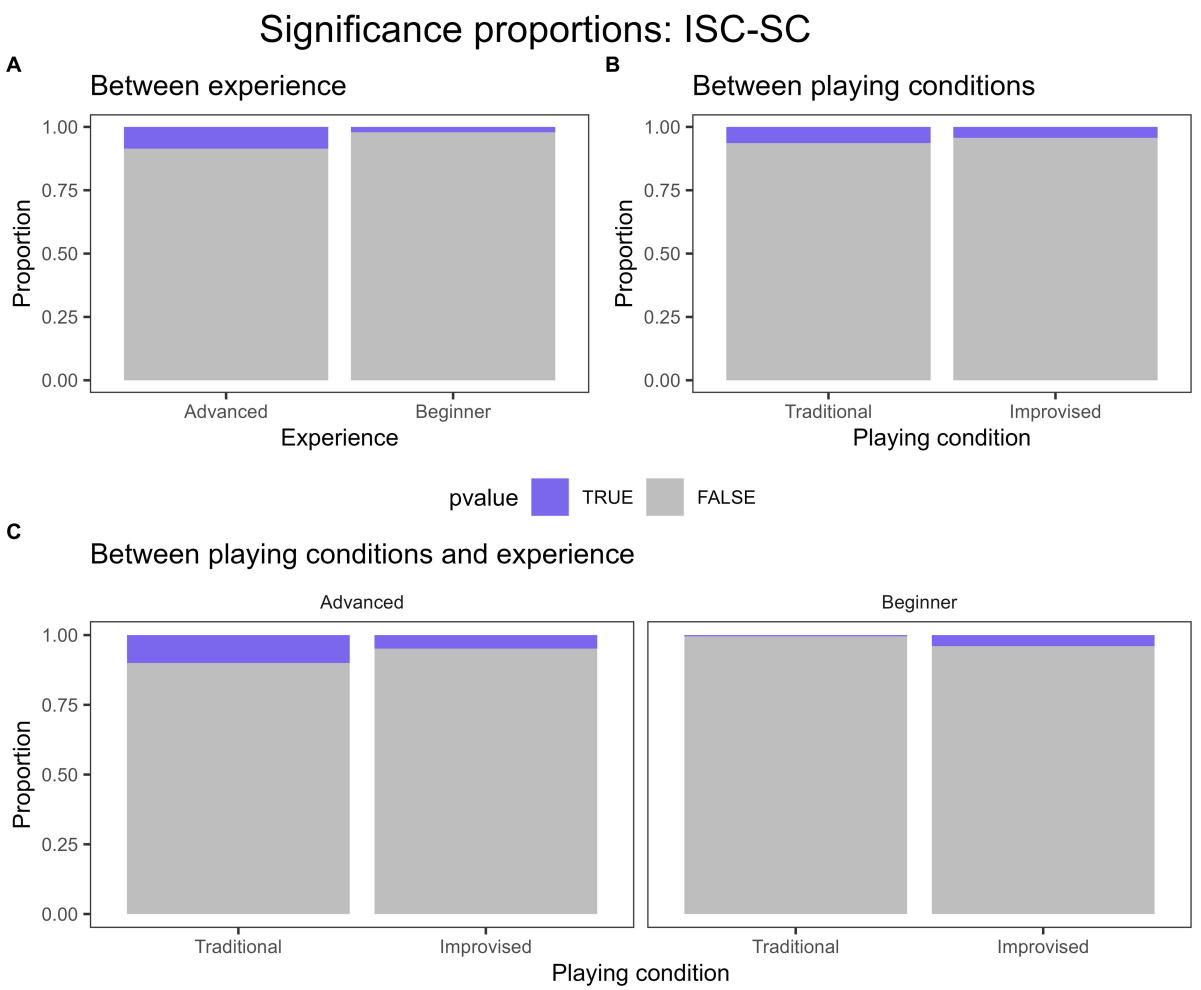
Proportion of significance for ISC-SC displayed in coloured regions for **(A)** between experience levels across both playing conditions, **(B)** between playing conditions across both experience groups, and **(C)** between both playing conditions and experience groups. Significance determined using FDR of 0.05.

For ISC-SC in traditional playing, there was a significant difference in the proportion of significant ISC-SCs between beginner and advanced experience groups (*p* < 0.001), a greater proportion of significant ISC-SC occurred for the advanced group than the beginner group in traditional playing. No significant difference in experience groups was found for improvised playing. We then split the data to isolate experience groups and account for differences between playing conditions within them. For beginner players, we found a significant difference in the proportion of significance between improvised and traditional playing (*p* = 0.015). Here, the proportion of significant ISC-SC seemed to be greater in improvised playing than in traditional playing for the beginner group. For the advanced group, a non-significant trend level difference between traditional and improvised playing was found for the advanced group (*p =* 0.078), whereby contrary to the beginner group, the proportion of significant ISC-SCs may have been slightly greater in traditional playing than in improvised playing. These results indicate that the proportion of significant ISC-SC values was greater overall for advanced players, at least in traditional playing, and that the proportion of significant ISC-SC between playing conditions when isolating beginner and advanced groups seemed to be opposing. Figure 3 displays these proportions of significance for ISC-SC graphically.

**Figure 4:**
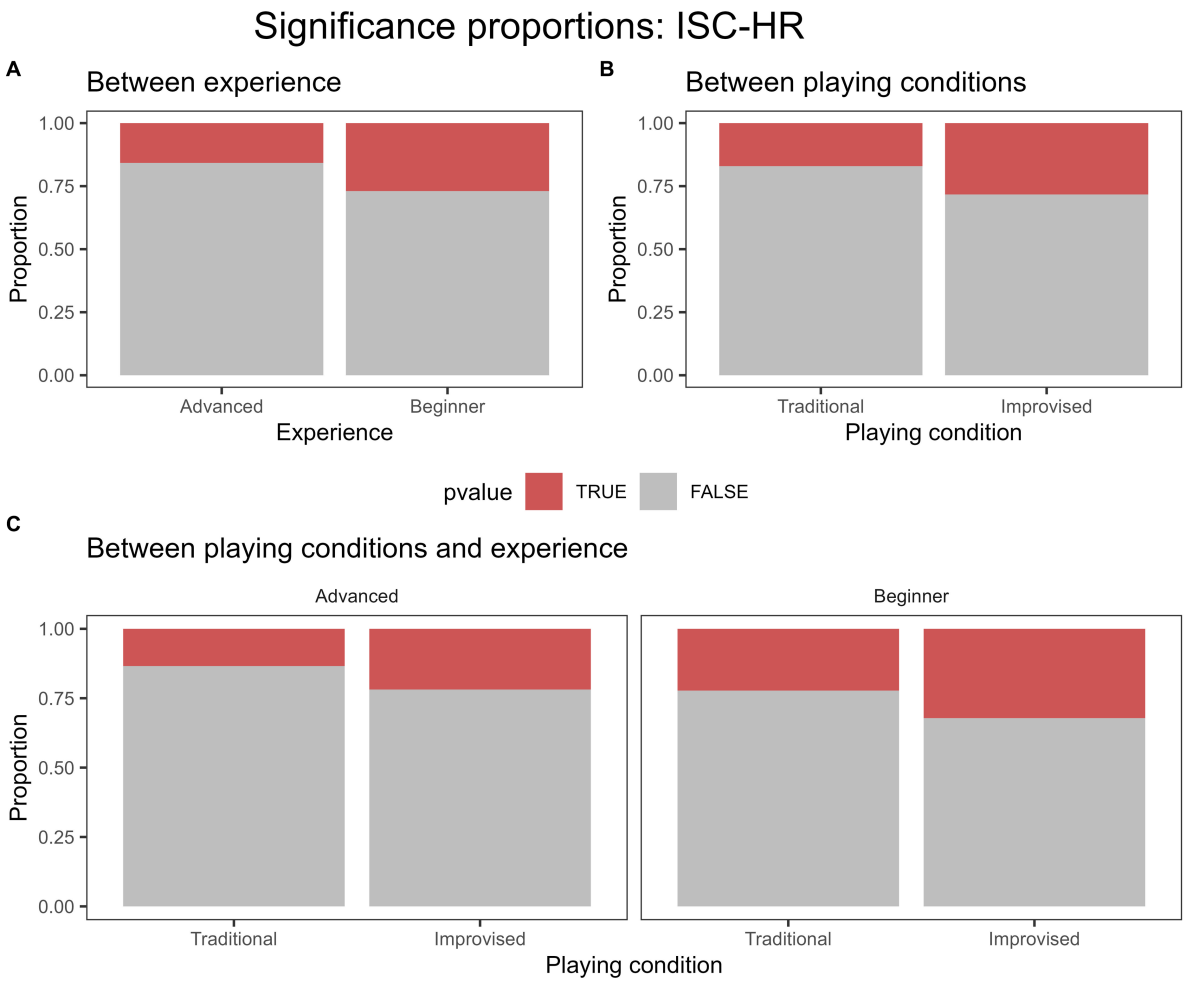
Proportion of significance for ISC-HR displayed in coloured regions for **(A)** between experience levels across both playing conditions, **(B)** between playing conditions across both experience groups, and **(C)** between both playing conditions and experience groups. Significance determined using FDR of 0.05.

For ISC-HR, we found a significant difference between the beginner and advanced experience groups and the proportion of significance in traditional playing (*p* = 0.006) and in improvised playing (*p* = 0.026). Within these, beginners yielded a greater proportion of ISCs across both playing conditions. We then split the data to account for differences between playing conditions for each experience group separately. Overall, the proportion of significant ISC-HRs was significantly greater in improvised playing than in traditional playing for both the advanced group (*p =* 0.03) and beginners (*p* = 0.009). These results suggest that the proportion of significant ISC-HR values was greater overall for beginners, and overall for improvised playing. Figure 4 displays these proportions of significance for ISC-HR. Average significant ISCs were also compared between groups and playing conditions to assess differences in values (see Supplementary material).

### 3.3. How does self-reported shared flow relate to average physiological synchrony in traditional and improvised playing?

For the following analyses, all significant and non-significant ISC values were averaged across all sections, providing overall measures of average physiological synchrony denoted by ISC-HR_mean_ and ISC-SC_mean_. Two separate linear mixed models were fitted for ISC-HR_mean_ and ISC-SC_mean_. For each of these, we investigated the fixed effects of playing conditions (improvised vs. traditional) and the shared flow factors, identified in response to the first research question of *Absorption* and *Awareness*. A random effect was only assigned experience level, as subject-level variance was negligible, and therefore did not warrant an additional subject-level random effect to explain the observed variance. Results for each indicated that the optimally fitting models involved an interaction between a shared flow factor and the playing condition.

#### 3.3.1. Dependent variable ISC-HR_mean_

**Figure 5:**
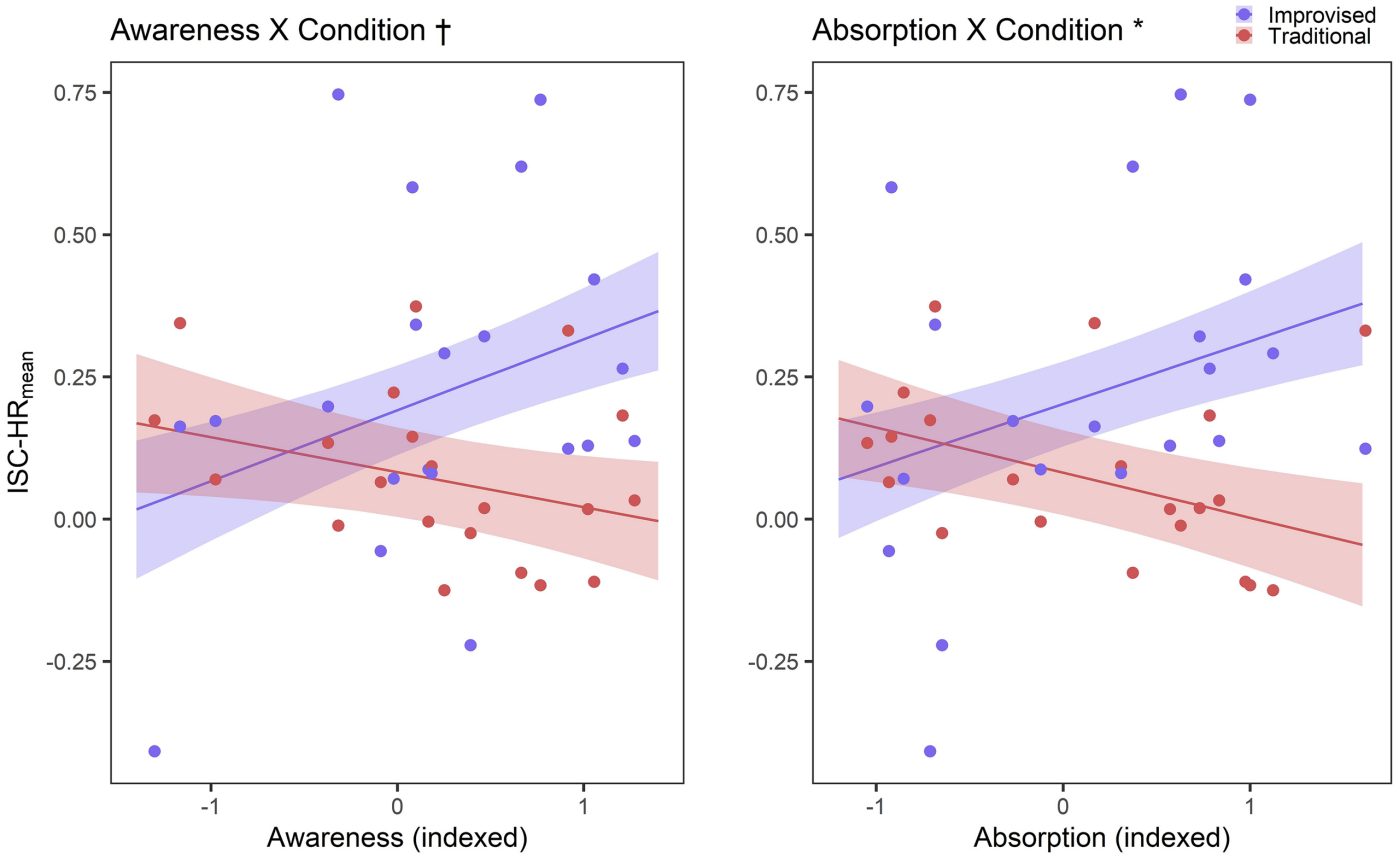
Results for RQ3. Graphical depiction of predictions of ISC-HR_mean_ by shared flow factors interacting with music condition. Shaded areas represent standard error. Predictions for ISC-HR_mean_ in improvised playing are represented in blue, while traditional playing is represented in red. † Denotes trend-level model fit improvement at *p* ≤ 0.10, *denotes significant model fit improvement at *p* < 0.05.

For the first mixed model comparisons, detailed model comparisons are listed in Table 2, while full fixed effects results for these most optimally fitting models are provided in Table 3. An interaction model revealed that ISC-HR_mean_ may be significantly predicted by shared *Absorption* as an overall fixed effect in improvised playing, though ISC-HR_mean_ may decrease with *Absorption* specifically within traditional playing. An interaction model with shared *Awareness* demonstrated a trend-level fit above that of the condition model, and the findings of this were similar to that of shared *Absorption*. ISC-HR_mean_ may be predicted by an overall fixed effect of shared *Awareness* in improvised playing, while ISC-HR_mean_ seems to decrease with shared *Awareness* for traditional playing. Graphical figures are provided in Figure 5. These graphical figures suggest that contrary to the traditional playing condition, there may be a positive association between ISC-HR_mean_ and both shared *Absorption* and *Awareness* for improvised playing.

**Table 2:** Dependent variable: ISC- HR_mean_; independent variables: condition (traditional, improvised), shared flow factors (*Absorption*, *Awareness*).

Model	AIC	BIC	Marginal *R*^2^	Conditional *R*^2^	Improvement in model fit	
					*X*^2^(1)	*p*
ISC- HR_mean_ Null Model	0.8	6.0		0.136		
ISC- HR_mean_ ~ Condition	−2.2	4.7	0.099	0.239	4.9991	0.02536
ISC- HR_mean_ ~ Condition + *Awareness*	−0.7	8.0	0.108	0.253	0.5083	0.4759
**ISC- HR**_**mean**_ **~ Condition × Awareness**	**−3.5**	**6.9**	**0.190**	**0.339**	**5.3242**	**0.0698°**
ISC- HR_mean_ ~ Condition + *Absorption*	−0.4	8.3	0.103	0.233	0.1569	0.0692°
ISC- HR_mean_ ~ Condition × Absorption	−4.4	6.0	0.207	0.342	6.1872	0.0453°

Linear mixed effects model between ISC-HR_mean_ values and shared flow factors. Condition is given as a fixed effect, and experience group as a random effect. Bold text indicates the most optimally fitting model. Null model: HR ~ 1 + (1|experience). As compared to HR ~ Condition. *X*^2^(1) increases to *X*^2^(2) for two fixed effects, and *X*^2^(3) in interaction models; HR: *N* = 21.

**Table 3:** Fixed effects results for the most optimally fitting interaction models with ISC-HR_mean_ as a dependent variable.

Dependent	Estimate	Std. Error	*df*	*t*	*p*
*ISC-HR_mean_ ~ Condition × Awareness + (1|experience)*					
(Intercept)	0.191	0.078	2.870	2.441	0.096^†^
Condition (traditional)	−0.109	0.062	39.993	−1.758	0.087^†^
Awareness	0.125	0.058	40.023	2.140	0.039*
Condition (traditional):Awareness	−0.186	0.082	39.993	−2.262	0.030*
*ISC-HR_mean_ ~ Condition × Absorption + (1|experience)*					
(Intercept)	0.202	0.075	2.784	2.707	0.080^†^
Condition (traditional)	−0.120	0.060	39.946	−2.010	0.051^†^
Absorption	0.110	0.053	40.424	2.073	0.045*
Condition (traditional):Absorption	−0.190	0.074	39.946	−2.551	0.015*

^†^Denotes non-significant trend at *p* ≤ 0.10, *denotes significance at *p* < 0.05. *N* = 21.

#### 3.3.2. Dependent variable ISC-SC_mean_

**Figure 6:**
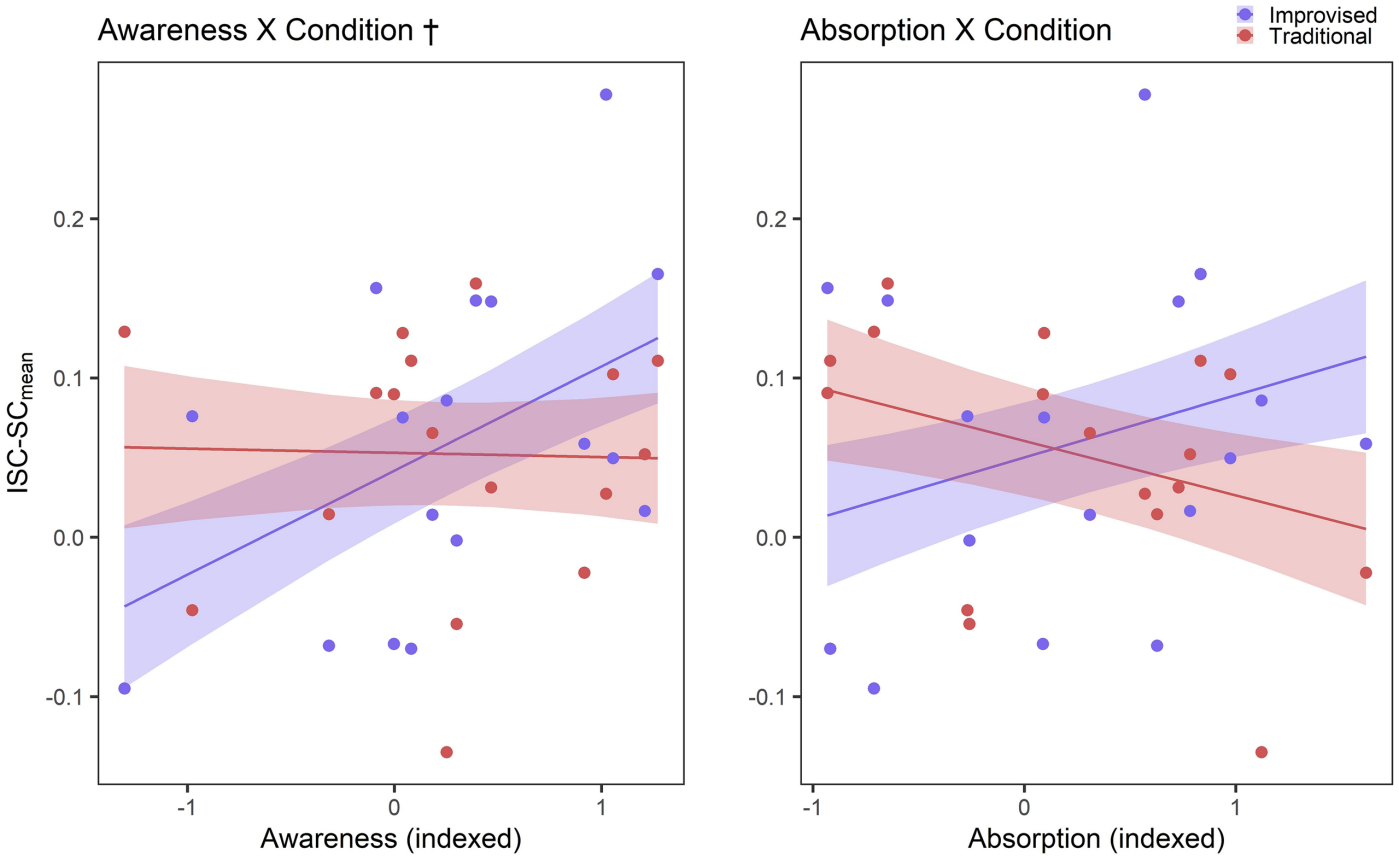
Results for RQ3. Graphical depiction of predictions of ISC-SC_mean_ by shared flow factors interacting with music condition. Shaded areas represent standard error. Predictions for ISC-SC_mean_ in improvised playing are represented in blue, while traditional playing is represented in red. † Denotes trend-level model fit improvement at *p* ≤ 0.10.

For the second mixed model comparisons, a comparable pattern emerged. Detailed model comparisons are listed in Table 4, while full fixed effects results for this model are provided in Table 5. An interaction model indicated that ISC-SC_mean_ may be predicted by an overall fixed effect of shared *Awareness* in improvised playing, while ISC-SC_mean_ decreases with *Awareness* on a trend level in traditional playing, in the most optimally fitting model. Figures illustrating these predictions between factors of playing condition, shared flow factors, and ISCs are provided in Figure 6. Graphical visualisation shows that while the relationship between shared *Awareness* ISC-SC_mean_ may be negligible in traditional playing, it seems to be positively associated in the context of improvised playing. Although the ISC-SC_mean_ interaction model with shared *Absorption* did not show any significant improvement in the model fit, the related figure is still included for the sake of consistency.

**Table 4:** Dependent variable: ISC-SC_mean_; independent variables: condition (traditional, improvised), shared flow factors (*Absorption*, *Awareness*).

Model	AIC	BIC	Marginal *R*^2^	Conditional *R*^2^	Improvement in Model Fit	
					*X*^2^(1)	*p*
ISC-SC_mean_ Null Model	−66.8	−62.2		0.201		
ISC-SC_mean_ ~ Condition	−64.8	−58.7	0.002	0.203	0.0639	0.800
ISC-SC_mean_ ~ Condition + Awareness	−65.5	−57.9	0.065	0.254	2.7614	0.251˄
**ISC-SC**_**mean**_ **~ Condition × Awareness**	**−67.0**	**−57.8**	**0.139**	**0.332**	**6.2524**	**0.100˄**
ISC-SC_mean_ ~ Condition + Absorption	−62.8	−55.2	0.002	0.207	0.0766	0.962˄
ISC-SC_mean_ ~ Condition × Absorption	−65.1	−56.0	0.098	0.309	4.3746	0.224˄

Linear mixed effects model between mean ISC values and shared flow factors. Condition is given as a fixed effect, and experience group as a random effect. Bold text indicates the most optimally fitting model. Null model: SC ~ 1+ (1|experience). ^^^As compared to SC Null Model. *X*^2^(1) increases to *X*^2^(2) for two fixed effects, and *X*^2^(3) in interaction models; SC: *N* = 17.

**Table 5:** Fixed effects results for the most optimally fitting interaction model with ISC-SC_mean_ as a dependent variable.

Dependent	Estimate	Std. Error	*df*	*t*	*p*
*ISC-SC_mean_ ~ Condition × Awareness + (1|experience)*					
(Intercept)	0.042	0.033	2.954	1.264	0.297
Condition (traditional)	0.011	0.026	31.995	0.424	0.674
Awareness	0.065	0.025	32.028	2.605	0.014*
Condition (traditional):Awareness	−0.068	0.035	31.995	−1.921	0.064^†^

^†^Denotes non-significant trend at *p* ≤ 0.10, *denotes significance at *p* < 0.05. *N* = 17.

## 4. Discussion

In furthering our knowledge of mechanisms of joint music-making, this exploratory study is the first of its kind to investigate the experience of shared flow and physiological correlates in the context of Javanese gamelan. The primary aims were to explore aspects of shared flow (RQ1) and synchronised physiological parameters of heart rate (HR) and skin conductance (SC) *via* inter-subject correlation (ISC) (RQ2) while participants played Javanese gamelan pieces. We assessed whether there might be any differences in significant mean ISCs between the experience levels of the players and between improvised or traditional pieces. Finally, we assessed the extent that the physiological synchrony was related to self-reported flow (RQ3). Shared flow has scarcely been studied in group music contexts. Where it has, potential physiological patterns underlying the experiences have not been measured. Additionally, gamelan has had little attention from psychologists and presents an opportunity to study such effects in a highly ecologically valid setting, where cyclicality and egalitarianism meet in instrumental form. Our findings revealed a complex picture of the differences in ISC-HR and ISC-SC underlying traditional playing and improvised playing between experience groups. Shared flow factors and ISCs were positively associated with each other for improvised playing, and negatively for traditional playing.

In addressing our first research question, we used the Shared Flow Scale (Zumeta et al., 2016) to explore aspects of shared flow. The SFS was initially selected for two reasons. First, the scale had previously been used in a comparable context of a drumming march at a festival. Second, shared flow has been identified as parallel to a Javanese concept of *ngeli* specifically in a gamelan ensemble. However, in its original form, our confirmatory factor analysis of the Shared Flow Scale (Zumeta et al., 2016) did not yield an admissible model. This could have been due to the situation. Shared flow experiences in situations of music-making might be fundamentally different to the classic nine-factor conceptualisation shared with Csikszentmihalyi’s (2000) flow. For that reason, it may be difficult to capture such an experience using similar scales. Instead, our solution encompassed factors that we labelled as *Awareness* and *Absorption*, reflecting both the absorbing and challenging nature of gamelan playing. We see these factors as potentially comparable to the need for simultaneous self-other integration and separation in group music performance, highlighted by Liebermann-Jordanidis et al. (2021). However, the SFS does not clearly distinguish between potential antecedents and outcomes of shared flow, and where antecedents may be equivalent across both shared interactive flow and group flow, the outcomes may differ (Hackert et al., 2022). Future work involving self-reports on shared flow experiences may therefore wish to explicitly differentiate between antecedents and outcomes through the use of separate scales or groups of items.

Our findings regarding the potential differences in both the proportion of significant ISCs and the overall value of significant ISCs were mixed. We found a greater proportion of significant ISC-SC for advanced players compared to beginner players, while the opposite was true for ISC-HR. When splitting the data by experience, the proportion of significant ISC-HR seemed to be greater for improvised playing than traditional across both experience groups, while for ISC-SC, the difference in the proportion of significance between the two playing conditions was opposing for each experience group, at least on a non-significant trend level. Potentially this could indicate differences in required effort associated with levels of expertise and resultant physiological parameters. Furthermore, beginners were taught their parts aurally, while many of the advanced players still relied on written notation. This distinction may have also accounted for these inconsistencies between measures.

HR indicates activity of both the sympathetic and parasympathetic branches of the autonomic nervous system, while SC is an indicator of sympathetic activity alone. Accordingly, the differences in findings between experience levels and playing conditions are not necessarily surprising. Levels of attention may not affect parasympathetic activity (Pérez et al., 2021), while the degree of challenge and associated effort facilitating flow experience might (Thissen et al., 2021). Further to this, sympathetic activation as an indicator of arousal appears to form an inverted U-shape relationship with flow (Peifer et al., 2014; Tozman et al., 2015). For that reason, it seems plausible that a greater proportion of significant ISC-HR was present for beginner players who may have felt more of a fluctuating shared sense of challenge overall. Nevertheless, when observing the differences between playing conditions for each experience group separately, improvised playing yielded a greater proportion of significant ISC-HR for both groups. This perhaps instead relates to improvisation leading to greater levels of togetherness, enhanced engagement, and enjoyment (Noy et al., 2015), which may also be supported by a greater proportion of significant ISC-SC in improvised playing compared to traditional playing for beginners. Meanwhile, the finding of a greater proportion of significant coupling in traditional playing for advanced players may be attributed to heightened arousal levels, in that their piece involved more temporal and structural changes than the beginner piece. These findings must be taken with great caution, however, as the number of significant data points for ISC-SC was far fewer than for ISC-HR, and the sample is small overall. As such, we acknowledge these findings are limited in their power and form merely a starting point in a novel area of research. There is a necessity to further disentangle the roles of shared sympathetic and parasympathetic activity in relation to shared flow experiences in differing contexts. Future studies with greater power may therefore find it worthwhile to explore such effects of expertise and playing style further.

Crucially, through the use of linear mixed effects models, we found that overall physiological synchrony underlying flow seems to be fundamentally different between improvised and traditional playing. The shared flow factors seemed to negatively predict mean ISCs in traditional playing, and positively predict mean ISCs in improvised playing, on at least a trend level. These findings support the notion that ISC might not only occur in situations involving joint listening settings (Czepiel et al., 2021; Dauer et al., 2021; Pérez et al., 2021), but also in joint music performance settings. Extending knowledge of how more successfully coordinated action may result in greater physiological coupling on some level, even for non-experts or those with a low level of in-group familiarity (Gordon et al., 2020; Ruiz-Blais et al., 2020), our results demonstrate a novel contribution of shared flow experiences. Tightly coordinated actions are necessary for the successful performance of music, which in turn is often found to involve some degree of shared flow experience (Cochrane, 2017; Tay et al., 2021; Magyaródi et al., 2022).

Contrary to a previous finding that did not find a relationship between shared flow and overall mean HR (Horwitz et al., 2021), we observed a relationship between mean physiological coupling and shared flow. As this relationship seems to differ between improvised and prescribed playing conditions, there seem to be differences between physiological behaviours underlying shared interactive flow and group flow. This finding may support the potential for improvised playing and associated flow to result in greater physiological connectedness. As Hackert et al. (2022) theorise, group flow arises from a context in which it relies on the interactivity of the ensemble. Shared interactive flow experiences are rather experienced on the individual level, as the occurrence of flow is not solely dependent on interactivity. The differences between these might be evident in the differences between traditional and improvised gamelan playing. In the former, many participants in the advanced ensemble were playing from notated music, especially the less experienced advanced players. Similarly, although the beginners were taught aurally initially, there was not enough time in the workshop dedicated to ensuring all players were aware of the inner workings of all parts with fluency. The resultant piece may have therefore been played successfully without much awareness towards the group, aside from the occasional tempo change or section change. For improvised playing, on the other hand, interactivity is fundamental to group improvisation, regardless of experience levels. Experience level was incorporated as a random effect in the linear mixed models due to mixed and minimal differences in the proportion of significant physiological synchrony between experience groups, and we did not acknowledge differences in learning style (i.e., from notation or memory) in our questionnaire. As such, these ideas are merely speculative, and we encourage future studies to explore the potential influence of learning style on the relationship between shared interactive flow, group flow, and underlying physiological synchrony.

## 5. Limitations

We emphasise here that almost all players in these ensembles were Western university students. The results may have looked quite different for more experienced gamelan players, especially those residing in Java who rehearse in more traditional ways. Something integral to the practise of gamelan is the focus on the group as a whole, rather than individual’s role within it. This notion may be difficult for Western players to fully resonate with, and therefore their engagement with traditional playing may still come from their Western ensemble experiences. Our advanced group, Gamelan Sekar Petak, is comprised of players who have been learning gamelan for many years, with some having studied in Java, alongside students who may have only been learning gamelan for less than a year at the university. Further to this, Gamelan Sekar Petak does not typically improvise in their rehearsals. It would, therefore, be fruitful to replicate the study with more experienced groups, groups in which members are comparably experienced, and/or groups that are more accustomed to improvisation. We anticipate that for groups with comparable traditional playing expertise to that of Gamelan Sekar Petak, and perhaps more experience with improvisation, similar results may arise. However, for more experienced gamelan groups, with comparably little improvisation experience, group flow experiences may be positively associated with physiological synchrony in the context of traditional playing. Subsequently, the degree to which our results are generalisable is unclear.

Our most prominent limitation is the small sample size of the study and unequal group sizes. In gamelan, the number of players is restricted due to the number of instruments, and thus the only way of increasing this sample is by studying the experience of multiple groups or multiple sessions with each group. The consequences of our sample comprising only two groups of varying experience levels, and recorded over three experimental sessions, may have had implications for every stage of the analysis. The beginner players were separated into two experimental sessions, also due to the number of instruments available, and the experience of these beginners may have been different between groups. The familiarity between group members was also not considered. Aside from differences in the experience of gamelan between the advanced group and the beginner group, they also differed in the amount of time in which they have been playing together and have known one another. Some beginner players may have known one another due to how they were recruited, but this was not accounted for in the data.

To tailor the potential for flow experiences to arise and improve the potential compatibility between participants’ skills and level of potential challenge, different pieces were selected for the beginner group and the advance group. The beginners’ piece was particularly repetitive and featured a short amount of musical material with only one melodic variant, and few tempo changes. This allowed players to learn the piece from memory in a short amount of time. To suit the ability of the advanced players, their piece was lengthier, with more variations to the structure and tempo and additional, more complex instruments. The differences in instrumentation, style, structure, and form of these pieces could have led to quite different flow experiences between groups. Furthermore, although the advanced players’ piece had been rehearsed for several months beforehand, many players were still reliant on the notation, while some had been familiar with the piece for years. Discrepancies in the learning style (i.e., aurally or written) were therefore present on both inter-and intragroup levels. Overall, a limitation of any quasi-experimental study design is that not all inter-individual differences, including and beyond those described, can be accounted for and controlled for. Future work may wish to improve experimental control, however as this is the first exploratory study in this area, its naturalistic study design necessitated compromises.

With regards to physiological measurement, SC sensors attached *via* the foot seemingly have not been used in an experiment of this kind before. Similar sensors monitoring SC attached to the hand were used to monitor shared physiological responses in response to a stimulus, rather than active activity, such as listening or group decision-making tasks (Czepiel et al., 2021; Dauer et al., 2021; Gordon et al., 2021). Furthermore, participants’ body hair was not removed to minimise discomfort and invasiveness as much as possible, and as a result, the preparation of the area for recording ECG was not at an optimum. To that end, much of the loss of data and suitable signals may be associated with participants’ continual movement and changing of sitting position, resulting in poor contact between the electrodes and the skin or disconnected leads.

Lastly, in an effort to improve ecological validity and reduce disturbance to the natural playing environment, questionnaires were only completed before and after the entire playing session, meaning any flow experience that differed between improvisation and traditional playing cannot be discerned. Further to this, the questionnaire was completed following the improvised session for both groups, and the experience of that may have been at the forefront of participants’ minds when responding to the SFS which asked for the overall experience of both traditional and improvised playing.

## 6. Conclusion

This study is the first to our knowledge that demonstrates the potential for shared flow experiences to be reflected by physiological synchrony and contributes to the sparse research into physiology in joint music-making. Importantly, in diversifying our understanding of music performance not just in Western music making, we explore ensemble performance in Javanese gamelan.

Within our study, two groups of differing levels of experience played traditional gamelan pieces and improvised as a group, while physiological parameters of SC and HR were continuously measured. After playing, participants completed a self-report measure of shared flow (SFS). We first assessed whether SFS is valid and reliable in the context of gamelan playing and proposed a potential two-factor solution. Following this, our findings surrounding differences in significant moments of physiological synchrony between levels of experience, and between traditional and improvised playing were unclear. However, we did find relationships between physiological synchrony and shared flow. More specifically we found positive associations between shared flow and average physiological synchrony within improvised playing and negative associations within traditional playing.

This finding may reflect the high degree of collectivism and collaboration required to participate in a group improvisation, whereas a group playing a traditional piece of gamelan music may still focus on their individual parts. Further studies may wish to reflect this potential difference in shared flow experience through a different self-report measure, assessing both antecedents and outcomes, as well as potential differences between individual- and group-level experiences of shared flow. Additionally, our findings are specific to the paradigm of Western participants playing Javanese gamelan music; future work in further understanding such mechanisms could be, for example, assessed in more hierarchical Western ensembles. These suggestions, in combination with our current findings, may provide valuable contributions towards a greater understanding of the experience of shared flow dynamics in ensemble music settings.

## Data availability statement

The raw data supporting the conclusions of this article will be made available by the authors, without undue reservation.

## Ethics statement

The studies involving humans were approved by the School of Arts and Creative Technologies Ethics Committee, University of York. The studies were conducted in accordance with the local legislation and institutional requirements. The participants provided their written informed consent to participate in this study.

## Author contributions

HG conceptualised and designed the study, carried out the experiments, conducted the formal data analysis, and created the full draft manuscript. HG and AC developed the scripts and visualisations. AC contributed to the formal analysis of the data and the development of the overall narrative, and edited and reviewed the manuscript. HE supervised the design of the study, advised on the analyses, and contributed to the review of the manuscript. All authors contributed to the article and approved the submitted version.

## Funding

At the time of publication, HG is under receipt of AHRC funding through the White Rose College of Arts and Humanities, grant number AH/R012733/1.

## Conflict of interest

The authors declare that the research was conducted in the absence of any commercial or financial relationships that could be construed as a potential conflict of interest.

## Publisher’s note

All claims expressed in this article are solely those of the authors and do not necessarily represent those of their affiliated organizations, or those of the publisher, the editors and the reviewers. Any product that may be evaluated in this article, or claim that may be made by its manufacturer, is not guaranteed or endorsed by the publisher.

## Footnotes

^1^In *balangun mlaku* style refers to a *gatra* with density of four notes, however *balangun nibani* style refers to a half-density *gatra* of two notes interspersed with rests, as described by Pickvance (2005, pp. 30-31). The pieces played in this study featured both styles, and so for the analysis, two *nibani* style *gatra* were equivocated with one *mlaku* style *gatra*, which were comparable in duration.
