# Supplementary material for: Physiological synchrony and shared flow state in Javanese gamelan: positively associated while improvising, but not for traditional performance
Source: Front Psychol. 2023 Aug 17;14:1214505. doi: 10.3389/fpsyg.2023.1214505 (PMC10469686; doi:10.3389/fpsyg.2023.1214505)
Supplement: Supplementary file 1 [file Data_Sheet_1.docx]

Supplementary Material: data sheet

# Section 3.2 Wilcoxon rank sum tests

The mean of all significant ISCs for each measure (ISC-SC­­_sig_ and ISC-HR_sig_) and each piece per participant was calculated in order to analyse and visualise differences in these values, and both beginner experiment groups were grouped together. As assumptions of normality were not met in the data, Wilcoxon rank sum tests were used to evaluate differences in the values of significant ISCs resulting from music conditions, and from experience groups. Detailed results are provided in Table 2. ISC-HR_sig_ across both experience groups was found to be higher in traditional playing (mdn=0.762) than in improvised playing (mdn=0.665). When isolating experience groups, the same was true for beginners, where ISC-HR_sig_ in traditional playing (mdn=0.950) was higher than improvised (mdn=0.893), and ISC-HR_sig_ for advanced players was higher in traditional (mdn= 0.721) than in improvised playing (mdn=0.648). The overall difference of ISC-HR_sig_ between experience groups across both playing conditions was also significant, whereby ISC-HR_sig_ for advanced players (mdn=0.684) was generally lower than ISC-HR_sig_ for beginner players (mdn=0.920).

For ISC-SC_sig ,_ far few significant values were found overall, particularly for beginners. Across both groups, it is suggested that there was greater ISC-SC_sig_ during improvised playing (mdn=0.590) than traditional playing (0.504). No significant difference was observed between playing conditions for beginners, and ISC-SC_sig_ was found to be only slightly higher for improvised playing (mdn=0.559) than in traditional playing (mdn=0.501) for advanced players on a trend level. Overall, ISC-SC_sig_ was significantly lower for advanced players (mdn=0.551) compared to beginners (mdn=0.645).

| **Table S1. Wilcoxon rank sum exact tests, estimating differences of mean ISC-HR_sig_ and ISC-SC_sig_ between music conditions (traditional and improvised playing) and experience groups.** | | | | |
| --- | --- | --- | --- | --- |
| Dependent | Factor | W | p | Effect size |
| ISC-HR_sig_ | Music condition (Beginners) | 0 | .02 | -0.774 |
|  | Music condition (Advanced) | 11 | .002 | -0.688 |
|  | Music condition (All) | 51 | .031 | -0.408 |
|  | Experience | 0 | <.001 | -0.941 |
| ISC-SC_sig_ | Music condition (Beginners) | 1 | 1 | 0 |
|  | Music condition (Advanced) | 36 | .082 | -0.463 |
|  | Music condition (All) | 46 | .093 | -0.419 |
|  | Experience | 0 | .017 | -0.598 |
| *Note:* ISC-HR_sig_ Advanced n=10, Beginners n=4, ISC-SC_sig ­_Advanced n=7, Beginners n=1. | | | | |


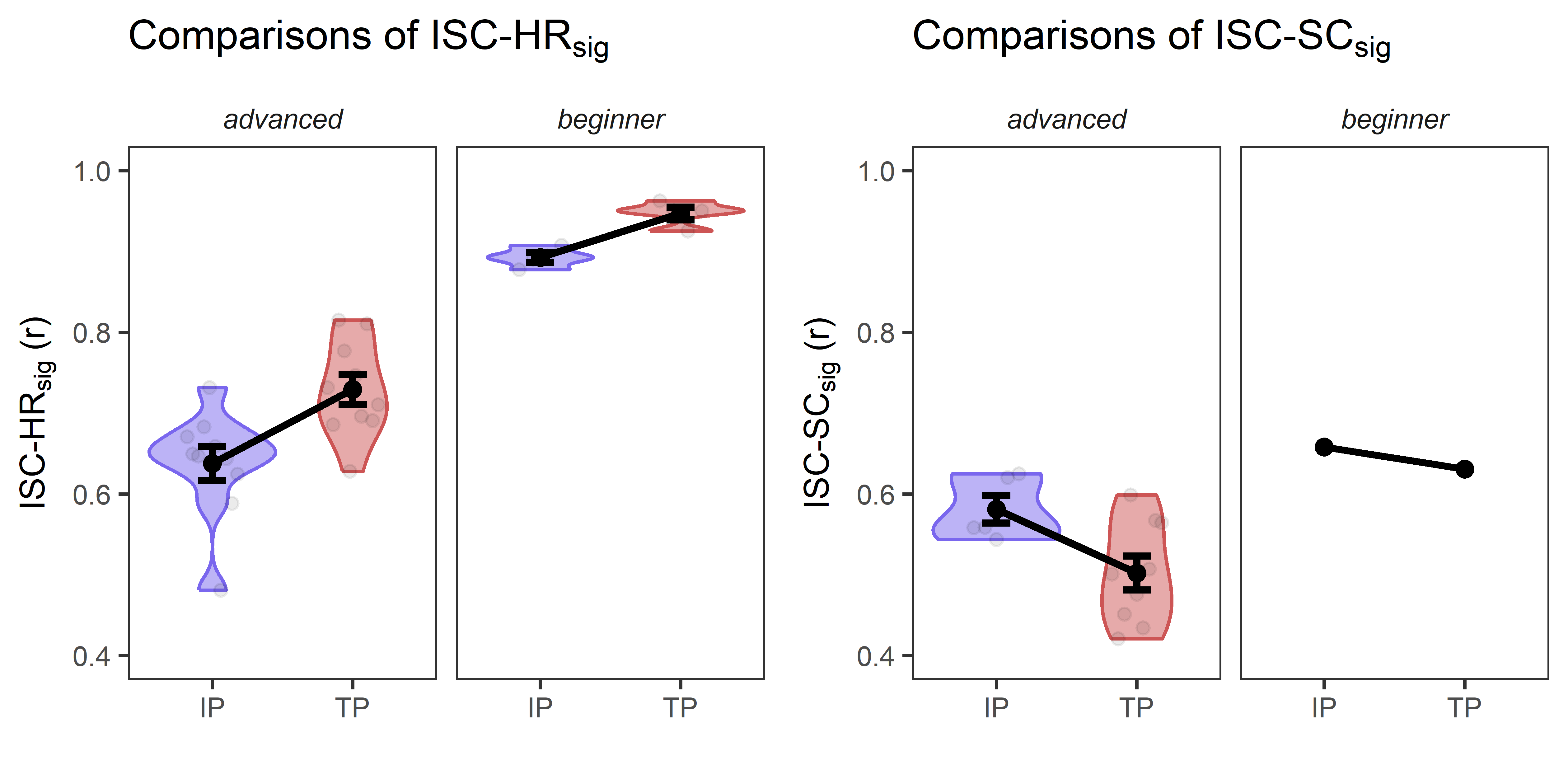


**Figure S1** Violin plots for ISC-HR _sig_ and ISC-SC _sig_ showing differences between music conditions of traditional playing (TP) and improvised playing (IP), grouped by experience levels.
